# Isolation and Characterization of Glycophorin from Carp Red Blood Cell Membranes

**DOI:** 10.3390/membranes4030491

**Published:** 2014-08-08

**Authors:** Takahiko Aoki, Kenji Chimura, Nobuhiro Nakao, Yasuko Mizuno

**Affiliations:** 1 Laboratory of Quality in Marine Products, Graduate School of Bioresources, Mie University, 1577 Kurima Machiya-cho, Tsu, Mie 514-8507, Japan; E-Mails: c.blacky@softbank.ne.jp (K.C.); t.nakano412@gmail.com (N.N.); 2 Toray Research Center, Inc., 1111 Tebiro, Kamakura, Kanagawa 248-8555, Japan; E-Mail: Yasuko_Mizuno@trc.toray.co.jp

**Keywords:** fish, red blood cell membranes, erythrocyte, glycophorin, oligosaccharide, sialic acid, *N*-glycolylneuraminic acid, bacteriostatic activity

## Abstract

We isolated a high-purity carp glycophorin from carp erythrocyte membranes following extraction using the lithium diiodosalicylate (LIS)-phenol method and streptomycin treatment. The main carp glycophorin was observed to locate at the position of the carp and human band-3 proteins on an SDS-polyacrylamide gel. Only the *N*-glycolylneuraminic acid (NeuGc) form of sialic acid was detected in the carp glycophorin. The oligosaccharide fraction was separated into two components (P-1 and P-2) using a Glyco-Pak DEAE column. We observed bacteriostatic activity against five strains of bacteria, including two known fish pathogens. Fractions from the carp erythrocyte membrane, the glycophorin oligosaccharide and the P-1 also exhibited bacteriostatic activity; whereas the glycolipid fraction and the glycophorin fraction without sialic acid did not show the activity. The carp glycophorin molecules attach to the flagellum of *V. anguillarum* or the cell surface of *M. luteus* and inhibited bacterial growth.

## 1. Introduction

Glycophorins and band-3 proteins found in mammalian [1,2] and avian [3,4,5,6] erythrocyte membranes are transmembrane glycoproteins. Glycophorins are a member of the sialoglycoprotein family and contain more sialic acid than band-3 proteins [7]. The glycophorins function as MN blood group antigens [8] or virus receptors [9,10]. Despite the importance of glycophorins to cell function and immunity, little is known about their function in teleost blood cells. 

We reported previously that some glycophorins, in the erythrocyte membranes of carp and rainbow trout, were stained with the periodic acid-Schiff’s (PAS) reagent [11]. However, it was difficult to estimate the apparent molecular mass using SDS-polyacrylamide gel electrophoresis (SDS-PAGE) gels, because the stain could not be clearly compared with that of human glycophorins. Similarly, PAS-stained avian glycophorin bands are also very faint on SDS-PAGE gels [12]. These issues highlight the need to isolate carp glycophorin from the red cell membranes to analyze its function.

The present paper describes the isolation and function of teleost glycophorins. We determined the sialic acid content and chemical composition of glycophorin isolated from carp erythrocyte membranes. In addition, we evaluated the physiological function of the carp glycophorin.

## 2. Materials and Methods

### 2.1. Materials

Live carp (*Cyprinus carpio)* were obtained from a local fish market. *N-*acetylneuraminic acid (NeuAc) was purchased from Wako Pure Chem. Ind. (Osaka, Japan). *N-*glycolylneuraminic acid (NeuGc) and human glycophorin were obtained from Sigma Chemical Co. (St. Louis, MA, USA). The GL-Pak Carbograph cartridge (300 mg/6mL) was purchased from GL Sciences Inc. (Tokyo, Japan).

The *Edwardsiella tarda* SU226 and *Vibrio anguillarum* ATCC19264 strains were a gift from Dr. I. Hirono, Tokyo University of Marine Science and Technology. The *Bacillus subtilis* ATCC 6633 strain was obtained from the Fisheries Research Institution in Aichi Prefecture. The *Aeromonas hydrophila* ATCC 7966 strain was obtained from the Institute of Medical Science, the University of Tokyo*. Micrococcus luteus* ATCC 9341 and *Escherichia coli* IFO3301 strains were cultured in our laboratory. The micro-organic media were purchased from Nissui Pharmaceutical Co. (Tokyo, Japan). All other reagents were analytical grade.

### 2.2. Preparation of Red Blood Cell Membranes

The erythrocyte membranes were prepared using the previous method with a slight modification [11]. The carp were anaesthetized with ethyl 3-aminobenzoate methanesulfonate (MS-222). Once anaesthetized, the blood was collected from the dorsal aorta using a heparinized syringe that was inserted through the mouth. The blood was diluted 1:1 with fish Ringer (145 mM NaCl, 5 mM CaCl_2_, 1 mM MgSO_4_, 4 mM KCl, 10 mM Hepes, and 5 mM glucose, pH 7.9) [13]. The diluted blood was placed on a Ficoll-Paque PLUS (GE Healthcare, Sweden), then centrifuged at 400× *g* for 40 min, and the red cells were collected. All subsequent procedures were carried out at 4 °C. The red cells were washed three times with fish Ringer, then hemolyzed by dilution in a 1:10–15 mixture of ice-cold 5 mM Tris-HCl (pH 7.6) containing 5 mM CaCl_2_ and 0.15 mM phenylmethylsulfonyl fluoride (PMSF). The suspension was placed on ice for 5 min and centrifuged at 40,000× *g* for 20 min. The upper precipitate layer was collected and suspended in ice-cold 5 mM Tris-HCl (pH 7.6) containing 0.15 mM PMSF, then centrifuged at 40,000× *g* for 20 min. The precipitate was collected and suspended in a two-fold dilution of Buffer A (75 mM Tris, 12.5 mM MgCl_2_ and 15 mM EDTA, pH 7.5) [13] containing 5 mM CaCl_2_ and 0.15 mM PMSF. The suspension was then homogenized with a tight-fitting Dounce homogenizer (10 strokes) and centrifuged at 40,000× *g* for 20 min. The resulting membrane pellet was re-suspended in Buffer A and homogenized (20 strokes). The membrane suspension was then placed on a sugar cushion (40% sucrose, 10 mM Tris-HCl and 10 mM MgCl_2_, pH 7.5) and centrifuged at 700× *g* for 15 min in a swing-out rotor. The overlay and inter-phase fractions were collected and centrifuged at 40,000× *g* for 20 min. The membrane pellets were re-suspended in Buffer B (20 mM Tris-HCl, 2 mM EDTA, pH 7.5) [13] and homogenized (10 strokes). The final membrane preparation was stored at −20 °C. Human erythrocyte membranes were prepared following the method of Hanahan and Ekholm [14].

### 2.3. Isolation of Glycophorin from the Red Blood Cell Membranes

Glycophorin from the carp red blood cell membranes was extracted using lithium 3,5-diiodosalicylate (LIS) and phenol based on a method that was developed for the extraction of human glycophorin [15]. The membrane preparation (*ca.* 40 mL) was first freeze-dried, and then 50 mM Tris-HCl buffer (pH 7.5) containing 0.3 M LIS was added to the dry membrane preparation up to *ca.* 25 mg·protein/mL. The preparation was homogenized with a tight-fitting dounce (10 strokes) then stirred at room temperature for 5–10 min. All subsequent procedures were carried out at 4 °C. Two volumes of distilled water were added to the homogenate followed by stirring for 30 min. The suspension was centrifuged at 45,000× *g* for 90 min. The supernatant was collected and mixed with an equal volume of freshly prepared 50% phenol in water. This suspension was stirred vigorously for 15 min and centrifuged at 4000× *g* for 1 h in a swinging-bucket rotor. The upper phase was collected and dialyzed against water. After dialysis, the inner solution was freeze-dried. The dry material was suspended with cold ethanol and stirred for 1–2 h, then centrifuged at 18,000× *g* for 20 min. The precipitate was washed with cold ethanol 3 times. The precipitate was dissolved in water, and then 2 drops of freshly prepared 5% streptomycin sulfate (pH 7.5) were added to aggregate the nucleic acids. After centrifugation at 10,000× *g* for 30 min, the supernatant was dialyzed against water for 24 h. Lastly, the dialyzed solution was centrifuged at 10,000× *g* for 30 min, and the resulting supernatant contained the glycophorin fraction.

### 2.4. SDS-Polyacrylamide Gel Electrophoresis (SDS-PAGE)

SDS-PAGE was performed with an 8.0%–9.0% separating gel and a 3.5% stacking gel [16]. The two-dimensional electrophoresis was carried out following the method developed by Tuech and Morrison [17]. One percent agarose gel was used to fix the disc gel to prepare a slab gel. The protein sample buffer was prepared following the method of Michel and Rudloff [13], except for those experiments to evaluate dimerization. The protein and glycoprotein bands were stained with Coomassie brilliant blue R-250 (CBB) and PAS reagent [18], respectively.

### 2.5. Miscellaneous Assays

The protein content was measured following the method of Lowry *et al.* [19] using bovine serum albumin as a standard. Nucleic acid concentrations were measured following the method of Croft-Lubran [20]. Sialic acid content was measured following the periodate-resorcinol method [21]. Total carbohydrate concentrations were determined using the phenol-H_2_SO_4_ method of Dubois *et al.* [22].

### 2.6. Amino Acid Analysis

The amino acid composition was measured using a MLC-203 amino acid auto analyzer (ATTO Corp., Tokyo, Japan) [23]. The proteins were hydrolyzed with 6 N HCl (constant boiling, Nacalai Tesque Inc., Kyoto, Japan) at 110 °C for 24 h. We used a standard protein solution (Amino Acids Standard Solution, Type H, Wako).

### 2.7. Preparation of the Carp Glycophorin Carbohydrate Fraction

The carbohydrate fraction was prepared by β-elimination according to the method of Carlson [24]. The carp glycophorin fraction was incubated in 1.0 M NaBH_4_ and 0.1 M NaOH in the dark at 37 °C for 48 h under N_2_ gas. The reaction was neutralized by careful addition of 1 M acetic acid at 0 °C. The mixture was centrifuged at 2500× *g* for 30 min, and the supernatant was evaporated. The concentrate was then dissolved in 5 mL of water and evaporated. This procedure was repeated three times. The preparation was then washed with water and evaporated to form a syrup that was washed with 5 mL methanol followed by 5 mL ethanol. The concentrated oligosaccharide alditol preparation, containing the carbohydrate fraction, was dissolved in 5 mL water.

### 2.8. High-Performance Liquid Chromatography (HPLC)

The high-performance liquid chromatography (HPLC) system consisted of a 625 LC system (a Waters 600E system controller and a 486 tunable absorbance detector) and a 741 data module (Waters Corporate, Milford, MA, USA).

The glycophorin fraction was eluted using a Superdex 200 HR 10/30 column (GE). The mobile phase consisted of 50 mM Tris-HCl buffer (pH 7.5) containing 0.1% SDS. The flow-rate was 0.5 mL/min. The sample volume injected into the column was 20 µL. The UV detector was set at 205 or 280 nm and 0.04 a.u.f.s. 

The carbohydrate fractions were eluted using a Glyco-Pak DEAE column (Waters). The mobile phase consisted of 10 mM Tris-HCl (pH 7.6). The column was eluted with a continuous linear gradient of 0–100 mM NaCl. The flow-rate was 1.0 mL/min. The sample volume injected into the column was 100 µL during each run. The UV detector was set at 205 nm. Peak fractions (P-1 and P-2) were pooled. We ran several repetitions of the HPLC procedure to increase the volume of each fraction. The oligosaccharide fractions were freeze-dried.

### 2.9. Desalting of Oligosaccharide Fractions

The oligosaccharide fractions were desalted using graphitized carbon [25]. Prior to use, the GL-Pak Carbograph cartridge column packing was washed with 40% (v/v) acetonitrile then rinsed with water. The carbon gel was swollen by storage in water for 12 h. The Carbograph column was washed with three bed volumes of 80% acetonitrile in 0.1% (v/v) TFA. After washing with water, the column was washed with 45% (v/v) acetonitrile in 2.5% (w/v) ammonium bicarbonate (one bed volume), rinsed with water, then washed with 80% acetonitrile in 0.1% (v/v) TFA followed by water and 1% NaCl. The freeze-dried oligosaccharide fraction was dissolved with 4 mL of 1% NaCl (*ca.* 10–20 µg carbohydrate/mL) and applied to the column. The column was washed with 5 bed volumes of water, then eluted with 45% acetonitrile in 2.5% ammonium bicarbonate. The eluate (*ca.* 30 mL) was pooled and evaporated.

### 2.10. Preparation of Sialic Acid and Hexosamine from Carp Glycophorin

Sialic acid was released from the glycophorin fraction by adding 5 mM HCl at 80 °C for 50 min under N_2_ gas. The reaction was terminated by adjusting the pH to 7.5 at 0 °C. The preparation was then dialyzed against water for 24 h. The inner solution contained the glycophorin fraction, which lacked sialic acid. The outer solution, containing the sialic acid, was evaporated and applied to a column of Dowex 2 × 8 (0.5 cm × 25 cm) in formate form. The column was washed with five bed volumes of water and then eluted with 1.1 M formic acid. The eluate was concentrated and used as the sialic acid preparation. The glycolyl groups in the preparation were determined using Eegriwe’s reagent [26].

The oligosaccharide fraction was hydrolyzed prior to preparation of hexosamine. Hydrolysis of the oligosaccharide fraction was conducted with 2.5 M trifluoroacetic acid at 100 °C for 6 h*.* The hexosamine fraction was then prepared based on the method of Boas [27] as follows. Following hydrolysis, the reaction mixture was evaporated and dissolved in water. The liberated hexosamine in the hydrolysate was adsorbed to a Dowex 50 × 2 column (1.0 cm × 25 cm) in H^+^ form. The column was washed with five bed volumes of water and eluted with 2 N HCl. The eluate was evaporated and the hexosamine content was measured following the method of Elson and Morgan [28].

### 2.11. Thin Layer Chromatography (TLC)

The sialic acid and hexosamine preparations were spotted on a TLC-plastic sheet silica gel 60 (Merck & Co., Inc., Darmstadt, Germany). The chromatography was carried out in ascending TLC using two solvents: *n*-propanol-water (7:3, v/v) for the sialic acid preparation and *n*-butanol-acetic acid-water (8:8:1, v/v) for the eluate containing hexosamine.

The sialic acids were visualized by spraying the plate with an orcinol reagent [26]. The hexosamines were visualized with a diphenylamine-aniline-phosphate reagent [29].

### 2.12. Microbiological Assay

The sensitivity test for the growth of several bacteria was performed using the disc-plate method. The agar medium containing bacterium (10^6–8^ cfu/10 mL medium) was layered on each plate medium and dried for 30 min. A paper disk (8 mm thick, Advantec Toyo Kaisha, Ltd., Tokyo, Japan) containing each fraction was placed on the medium and incubated at 20 °C. After 24–48 h, the inhibition zone was observed on each plate. For *E. tarda*, the inhibition zones were observed over a light box to discern the production of H_2_S. The composition of the plate media were as follows; *B. subtilis* and *M. luteus*, 3.8% sensitivity disk agar-N “*Nissui*”; *E. tarda*, 6% SS Agar “*Nissui*” containing 1% mannitol; *V. anguillarum*, 2.5% heart infusion broth “*Nissui*” containing 1.5% agar and 1.5% NaCl; other bacteria, 2.5% heart infusion broth “*Nissui*” containing 1.5% agar.

### 2.13. Electron Microscope

The interaction between carp glycophorin and *V. anguillarum* was examined by titrating the glycophorin on a glass cover slip treated with poly-l-lysine. After 20 min, the cover slip was dried in liquid carbon dioxide using a Hitachi HCP-2 critical-point drying apparatus (Hitachi Ltd., Tokyo, Japan). The cover slip was coated with platinum-palladium in a JEOL JFC-1100 ion-sputtering apparatus (JEOL) and observed under a JEOL JSM-T200 (SEM) operated at 10 kV.

The interaction of the carp glycophorin and *M. luteus* was observed by a carbon rod (5 mmØ carbon rod, JEOL) on a cover slip, which was treated with poly-l-lysine. After 40 min, the carbon plate was fixed with osmium tetroxide and stained to prevent the accumulation of a charge with tannic acid. The fixed carbon plate was dried in liquid carbon dioxide using a Hitachi HCP-2 critical-point drying apparatus. The carbon plate was then coated with platinum-palladium in a JEOL JFC-1100 ion-sputtering apparatus and observed under a Hitachi S-4000 (SEM).

The glycophorin was negatively dyed using 2% phosphotungstic acid (pH 7.0) and observed under a Hitachi H-800 (TEM).

## 3. Results

### 3.1. Isolation and General Properties of Carp Glycophorin Molecule

We obtained *ca.* 60 mg carp erythrocyte membrane protein from 20 mL of carp blood. The extraction process yielded *ca.* 4.0 mg glycophorin per gram of lyophilized carp erythrocyte membrane. The composition of the carp glycophorin preparation is given in Table 1. The amino acid composition of carp glycophorin is shown in Table 2, and it was noted that there was not a striking difference compared to that of human glycophorin A, with the exception of valine, lysine and arginine.

**Table 1:** Chemical composition of carp glycophorin (g/100 g).

Component	Carp	Human *^1^	Human *^2^
Protein	34.2	21.4	45.1
Hexose	55.2	26.4	12.2
Sialic acid	9.84	26.9	25.2
Hexosamine	0.78	25.2	16.2

*^1^ [30]; *^2^ [31].

**Table 2:** Amino acid composition of carp glycophorin (in moles percent).

Amino acid	Carp	Human *^1^	Human *^2^
Aspartic acid	7.25	8.90	6.7
Threonine	6.01	8.95	11.6
Serine	9.98	11.6	15.2
Glutamic acid	10.4	11.0	11.6
Proline	6.08	2.65	7.8
Glycine	6.01	8.49	4.8
Alanine	5.23	8.11	5.4
Valine	4.89	9.99	7.7
Methionine	1.32	0.14	1.3
Isoleucine	2.88	4.67	5.6
Leucine	5.26	8.60	5.3
Tyrosine	1.85	1.58	3.0
Phenylalanine	2.15	2.47	1.5
Lysine	14.7	5.12	4.0
Histidine	2.29	3.57	3.9
Arginine	8.72	3.97	4.8

*^1^ [30]; *^2^ the values are based on Table 2 of [31].

**Figure 1:**
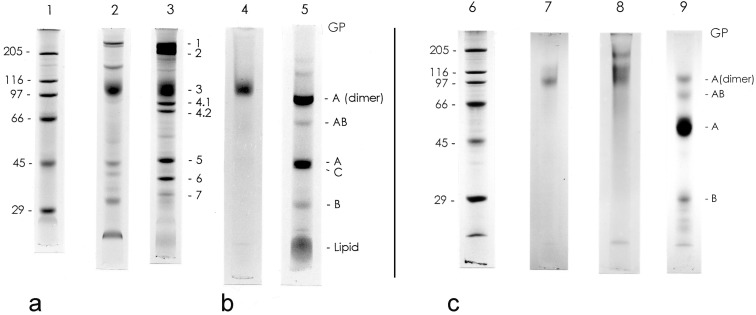
SDS-polyacrylamide gel electrophoresis of carp and human erythrocyte membranes and glycophorins. (**a**) Coomassie brilliant blue R-250 (CBB)-stained carp and human erythrocyte membranes. Lane 1, molecular mass standards: myosin (205 kDa); β-galactosidase (116 kDa); phosphorylase b (97 kDa); bovine albumin (66 kDa); egg albumin (45 kDa); carbonic anhydrase (29 kDa); Lane 2, carp erythrocyte membranes; Lane 3, human erythrocyte membranes. (**b**) periodic acid-Schiff’s (PAS)-stainedcarp and human erythrocyte membranes. Lane 4, carp erythrocyte membranes; Lane 5, human erythrocyte membranes. (**c**) CBB- and PAS-stained carp and human glycophorins. Lane 6, CBB-stained molecular mass standards (see Lane 1 for the order); Lane 7, CBB-stained carp glycophorin; Lane 8, PAS stained carp glycophorin; Lane 9, PAS stained human glycophorin. GP, glycophorin. The number denotes the membrane protein designations for human erythrocyte membrane proteins. Approximately 50 µg of membrane protein were applied/tube. A current of 3 mA was supplied/tube at room temperature.

**Figure 2:**
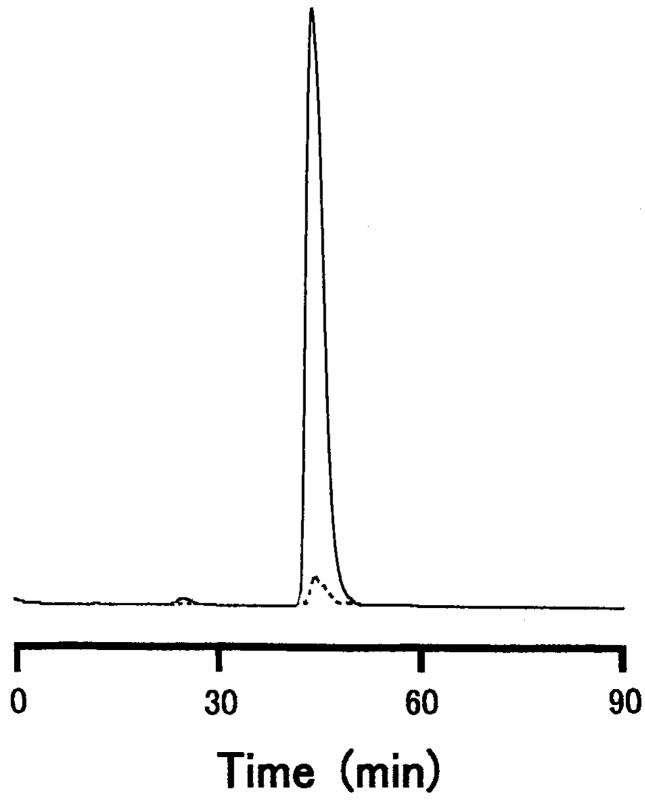
Chromatogram of carp glycophorin on a Superdex 200 HR 10/30 column. Five micrograms of carp glycoprotein were treated with the protein sample buffer [13] and injected into a Superdex 200 HR 10/30 gel filtration column. The glycoproteins were monitored at 205nm (—) and 280nm (•••).

The PAS-stained erythrocyte membrane preparation yielded one major band and some very faint and diffuse bands with higher and lower molecular weights (Figure 1, Lane 4), and the CBB stain yielded one diffuse protein band in the glycophorin preparation (Figure 1, Lane 7). In contrast, the PAS stained glycophorin preparation yielded two major bands (Figure 1, Lane 8). The intensity of the upper band of carp glycophorin preparation was greater after isolation from the cell membranes. The PAS stain pattern on the SDS-gels suggests that the carp erythrocyte membrane has fewer forms of glycophorin than human erythrocyte membrane. The main carp glycophorin was located near the position of the carp and human band-3 proteins (Figure 1a,b). In addition, the main carp glycophorin was positioned near the human glycophorin A (dimer) (Figure 1b,c). A lipid band, caused by contamination, that occurs close to the front of the PAS-stained gels [32] was absent from the carp glycophorin preparation. A single glycoprotein peak was detected using HPLC (Figure 2). Another minor peak at 205 nm appeared to be a dimer of the main glycophorin based on the retention time and SDS-PAGE banding (Figure 1c).

**Figure 3:**
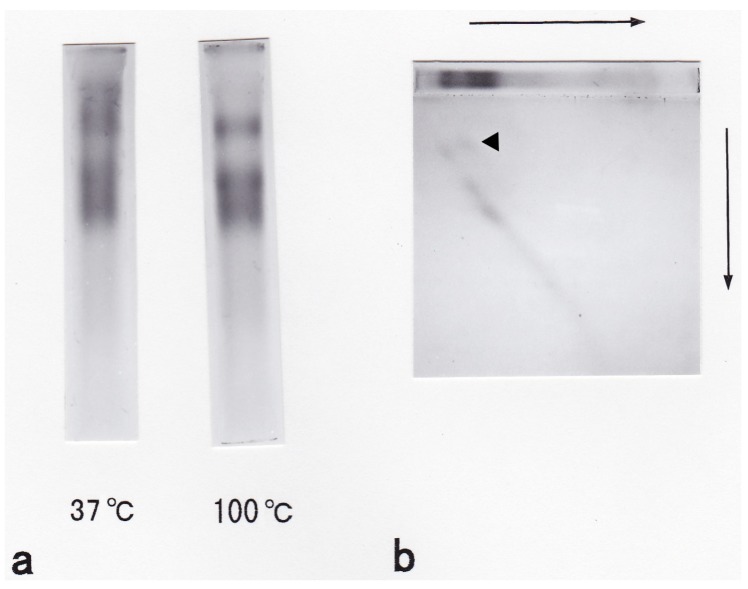
Dimerization of glycophorins separated by SDS gel electrophoresis. (**a**) Separation by SDS gel electrophoresis after treatment at 37 °C for 20 min or 100 °C for 3 min. The gels were stained with PAS stain. (**b**) Separation by two-dimensional electrophoresis. A portion of the gel was fixed and stained after the first separation and is positioned above the two-dimensional gel for reference. The gels were stained with PAS stain. A current of 15 mA was supplied/slab at room temperature.

To check for the dimerization of glycophorin, the preparation procedure prior to SDS-PAGE was modified. When the glycophorin preparation was treated at 37 °C, the upper band on the SDS-polyacrylamide gel was denser than when the preparation was treated at 100 °C (Figure 3a). The results of two-dimensional electrophoresis revealed that one portion of the main glycophorin band (indicated by the arrow in Figure 3b) was aggregated to appear as the upper band. Compared with the molecular standards on the SDS-polyacrylamide gel, the aggregated form was a dimer of the main carp glycophorin (Figure 1c).

### 3.2. Carbohydrate Moiety

**Figure 4:**
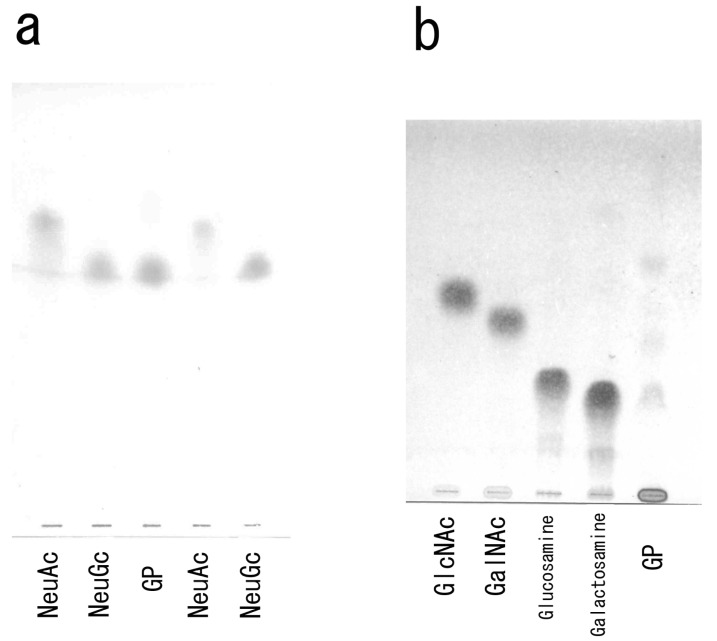
TLC of sialic acid and hexosamine. (**a**) Determination of sialic acid. GP, sialic acid from the carp glycophorin. (**b**) Determination of hexosamine. GP, hexosamine from the carp oligosaccharide hydrolysate.

The TLC analysis suggested that the glycophorin preparation contained the NeuGc form of sialic acid (Figure 4a). Similarly, the glycolyl group in the sialic acid preparation was identified as NeuGc using Eegriwe’s reagent. Using TLC, the hexosamine obtained from the carp glycophorin hydrolysate was identified as galactosamine (Figure 4b).

**Figure 5:**
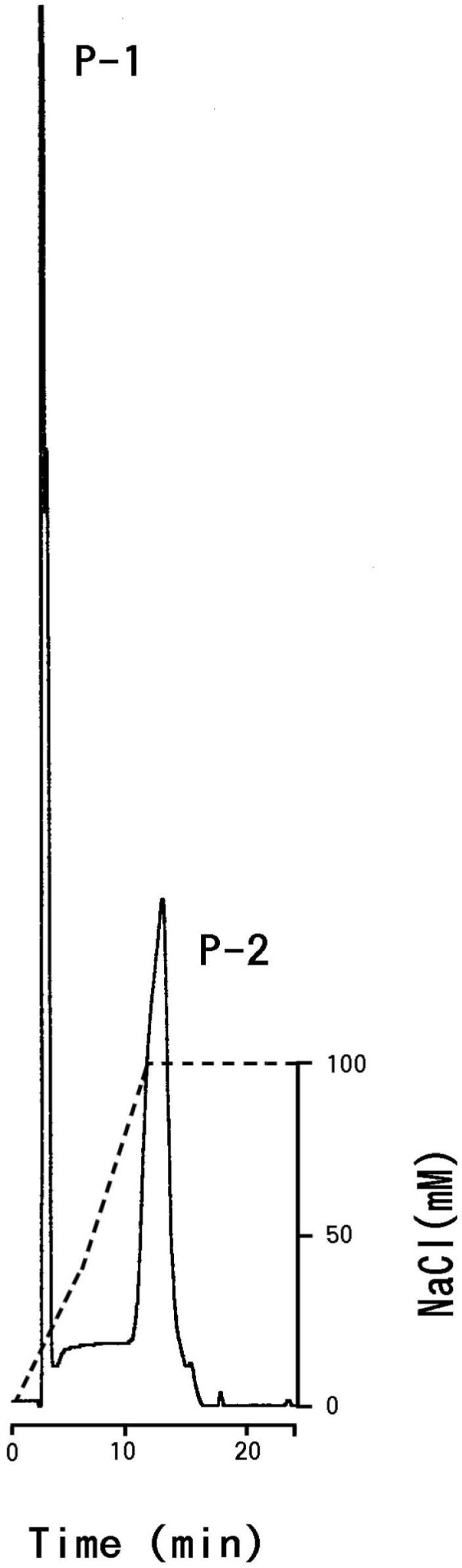
Chromatogram of carbohydrate preparation on a Glyco-Pak DEAE column. The oligosaccharides were monitored at 205 nm (—).

The chromatogram obtained during the HPLC run indicated that the carbohydrate fraction was separated into two peaks (P-1, P-2; Figure 5) Based on the chromatogram obtained using a NeuAc oligomer (α,2→8) kit (Nacalai Tesque, Inc.), P-1 contained one sialic acid residue, whereas P-2 contained two residues.

We obtained *ca.* 190 µg of P-1 and *ca.* 7.0 µg of P-2 (total carbohydrate) by an HPLC from carp glycophorin (*ca.* 4.0 mg protein). Using the graphite carbon column, the yield of desalted oligosaccharide was satisfactory (P-1: *ca.* 90% and P-2: *ca.* 100%).

### 3.3. Bacteriostatic Action of Carp Glycophorin

**Figure 6:**
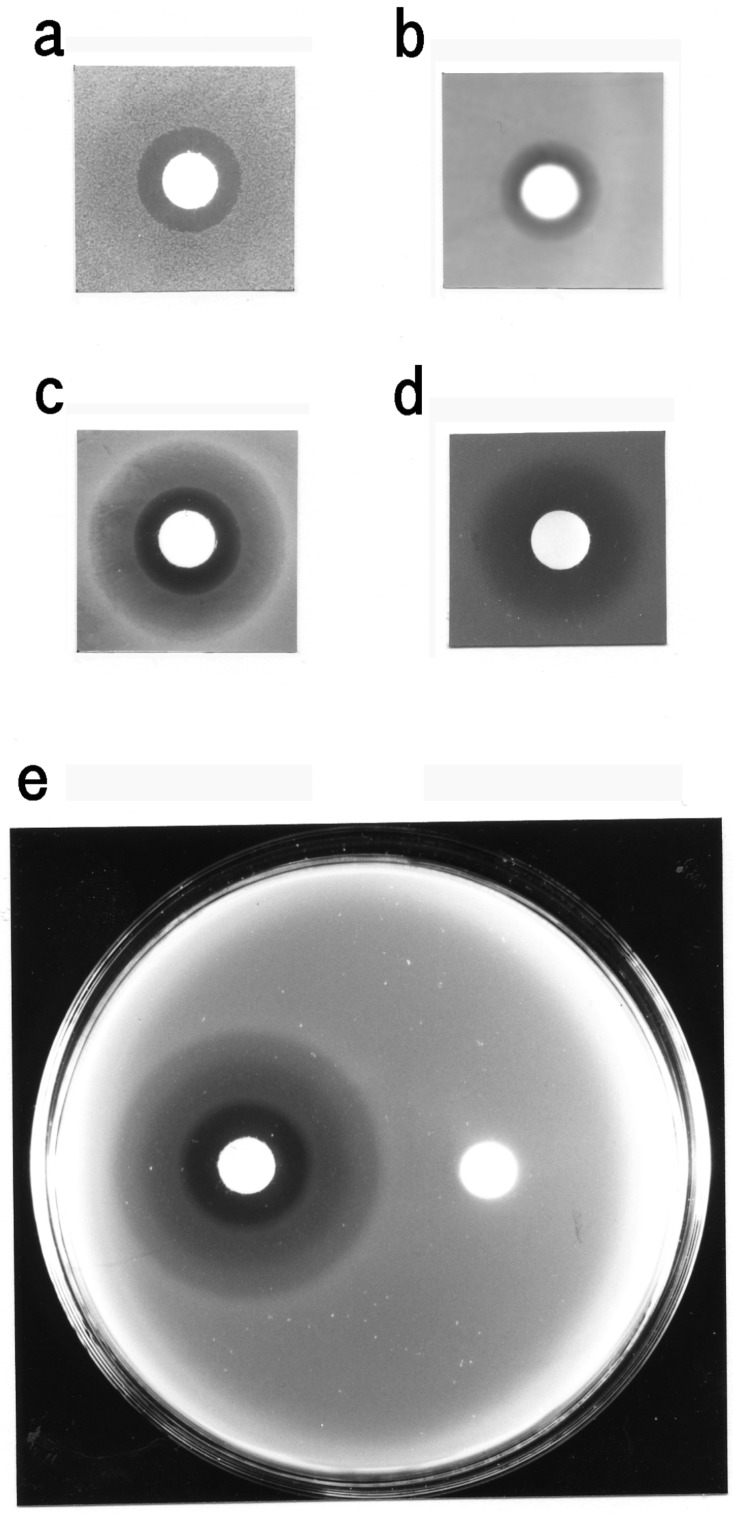
Sensitivity test for the growth of several bacteria by the disc-plate method. (**a**) Glycophorin fraction (*ca.* 15 µg·protein/disc) to *B. subtilis*; (**b**) glycophorin fraction (*ca.* 15 µg·protein/disc) to *A. hydrophila*; (**c**) glycophorin fraction (*ca.* 15 µg·protein/disc) to *M. luteus*; (**d**) glycophorin fraction (*ca.* 15 µg·protein/disc) to *V. anguillarum*; (**e**) glycophorin fraction (*ca.* 15 µg·protein/disc) to *E. coli* (left disc; glycophorin fraction, right disc; control).

**Figure 7:**
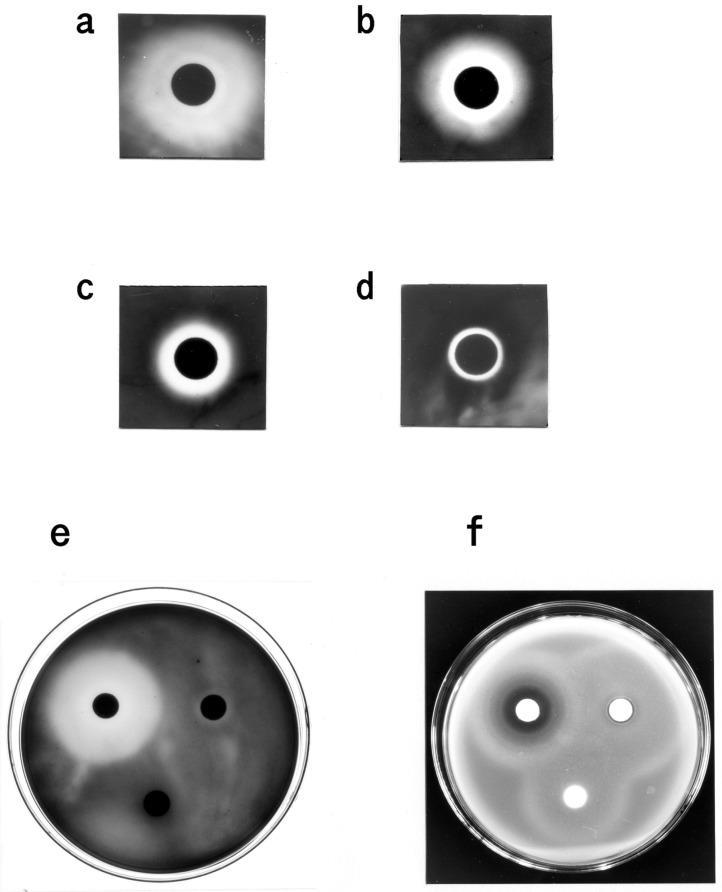
Sensitivity test for the growth of *E. tarda* and *M. luteus*. (**a**) Carp erythrocyte membranes (*ca.* 5 mg·protein/disc); (**b**) glycophorin fraction without streptomycin treatment (*ca.* 17 µg·protein/disc); (**c**) glycophorin fraction (*ca.* 15 µg·protein/disc); (**d**) carbohydrate fraction from carp glycophorin (*ca.* 4 µg/disc); (**e**,**f**) P-1 and P-2 fractions (*ca.* 8 µg/disc each); upper left disc, P-1; upper right disc, P-2; lower disc, control. (**a**–**e**) The plates containing *E. tarda*; (**f**) the plate containing *M. luteus*.

All of the test bacteria formed inhibition zones around the paper disc containing the glycophorin fraction (Figure 6). The erythrocyte membranes and the glycophorin fraction without streptomycin treatment inhibited bacterial growth (Figure 7a,b). The glycophorin, carbohydrate and P-1 fractions also exhibited bacteriostatic activity (Figure 7c–f). In contrast, the inhibition zones were not observed on the test bacteria plates by using the glycophorin fraction that lacked sialic acid or the human glycophorin. Similarly, by using the glycolipid fraction, extracted with chloroform-methanol (2:1, v/v) from the carp erythrocyte membranes, no inhibition zone was observed (data not shown). These results suggest that the test bacteria are sensitive to the monosialyl oligosaccharide from carp glycophorin (P-1).

**Figure 8:**
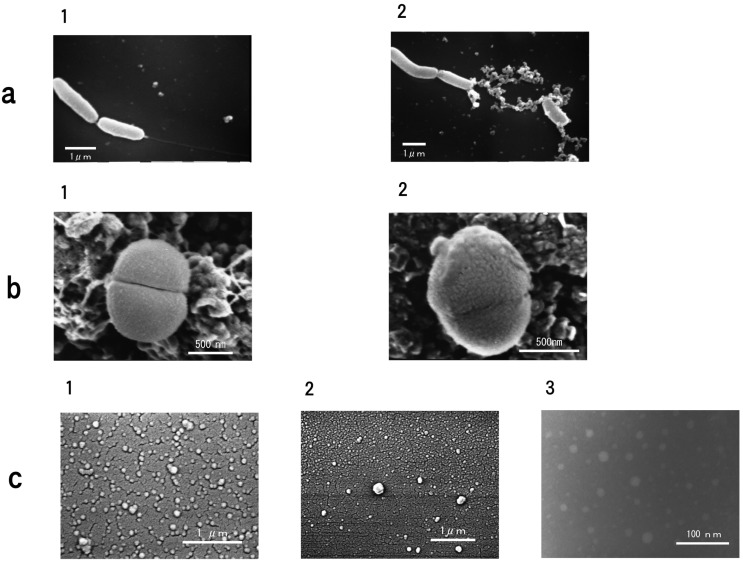
Electron microscope images of the bacteria and carp glycophorin. (**a**) *V. anguillarum* and carp glycophorin. 1, *V. anguillarum* without carp glycophorin; 2, *V. anguillarum* with carp glycophorin. An equal volume of glycophorin solution (*ca.* 0.4 µg·protein/20 µL) was added to the cell suspension (*ca.* 3 × 10^6^ cfu/20 µL) at 25 °C. (**b**) *M. luteus* and carp glycophorin. 1, *M. luteus* without carp glycophorin; 2, *M. luteus* with carp glycophorin. An equal volume of glycophorin solution (*ca.* 0.4 µg·protein/20 µL) was added to the cell suspension (*ca.* 3 × 10^6^ cfu/20 µL) of at 25 °C. (**c**) Carp glycophorin. 1, SEM image under the same conditions of *V. anguillarum* with carp glycophorin; 2, SEM image under the same conditions of *M. luteus* with carp glycophorin; 3, TEM image.

Based on the observations with the electron microscope, the carp glycophorin molecules attach to the flagellum of *V. anguillarum*, rather than the cell itself (Figure 8a). Conversely, the glycophorin molecules attach to the cell surface and cleavage line on *M. luteus* (Figure 8b). Carp glycophorin exists in molecular form and has a diameter of 40~220 nm from TEM images (Figure 8c-3).

## 4. Discussion

Michel and Rudloff reported the presence of major membrane protein bands on an SDS-PAGE gel derived from rainbow trout erythrocyte membranes. The bands were similar to those from mouse erythrocyte membranes, with the exception of bands 4.1 (weakly detected) and 4.2 (absent) [13]. We also found that the banding pattern of carp erythrocyte membranes was similar to that of human erythrocytes, again with the exception of bands 4.1 and 4.2. This is also consistent with observations in chicken [3] and pigeon [4,5] showing that band 4.2 proteins are poorly stained on SDS-gels.

The procedure for extracting glycophorin from chicken erythrocytes [6] was ineffective for the isolation of carp glycophorin. Therefore, we adopted the method developed by Marchesi and Andrews for human erythrocytes [15]. However, this procedure also extracted nucleic acids, which contaminated the membrane preparation. To remove the nucleic acids, we added streptomycin to the glycophorin solution and introduced a dialysis step. Although protein recovery decreased, the nucleic acid content of the preparation was below 3%.

The PAS-stained glycophorin preparation yielded two bands and one very faint and diffuse band that had a lower molecular mass. The upper glycophorin band was more intense than that from the carp erythrocyte membrane preparation.

To determine the dimerization of carp glycophorin, we performed a series of experiments following the methods of Tuech and Morrison [17]. Our results suggested that the upper glycophorin band on the SDS-PAGE gel was a dimer of the main band. We did not detect another glycoprotein with lower molecular mass following gel filtration and two-dimensional SDS-PAGE. Therefore, we concluded that the final glycophorin preparation consisted of a single glycophorin. However, our results did not confirm that the main glycophorin band was a dimeric form, as is the case with human glycophorin A [33].

There are several reports on the sialic acid component of both human and non-human sources of glycophorin. In human, glycophorin contains NeuAc, whereas the presence of NeuGc has been reported in horse [34], bovine [35], porcine [36] and monkey [37] glycophorin. However, to date, little is known about sialic acid in teleost glycophorins. This may be due to the difficulties associated with the preparation of fish erythrocyte membranes.

In the present study, NeuGc was detected in the carp glycophorin fraction by TLC and a colorimetric method. The results of additional HPLC experiments suggested that the sialic acid of carp glycophorin was NeuGc (data are not shown). Interestingly, NeuGc has also been reported in the eggs of rainbow trout, chum salmon and land-locked cherry salmon [38].

The carbohydrate fraction of carp glycophorin contained at least two kinds of O-linked oligosaccharides (P-1, P-2). The value of hexosamine was very low compared to the human composition (Table 1).

The carp glycophorin fraction exhibited bacteriostatic actions against several bacteria, including fish pathogens. The sensitivity test, using the carp erythrocyte membranes and the glycophorin fraction without streptomycin treatment suggested that streptomycin was not responsible for the bacteriostatic action. The bacteriostatic activity of the carbohydrate and oligosaccharide fractions suggested that the monosialyloligosaccharide of carp glycophorin (P-1) was responsible for mediating these activities.

## Abbreviations

NeuGc: *N*-glycolylneuraminic acid
Ac: acetyl
Fuc: fucose
Glc: glucose
Gal: galactose
GalNAc-ol: *N*-acetylgalactosaminitol
